# Arsenic exposure assists ccm3 genetic polymorphism in elevating blood pressure

**DOI:** 10.18632/oncotarget.23518

**Published:** 2017-12-21

**Authors:** Yanfang Gao, Zhiqiang Zhao, Linqing Yang, Xinxia Liu, Xiumei Xing, Huimin Zhang, Jianpei Yun, Xiaoyan Ou, Xiaolin Su, Yao Lu, Yi Sun, Yarui Yang, Jun Jiang, Dong Cui, Zhixiong Zhuang, Yun He

**Affiliations:** ^1^ Guangzhou Key Laboratory of Environmental Pollution and Risk Assessment, Sun Yat-sen University School of Public Health, Guangzhou, Guangdong 510080, China; ^2^ Shenzhen Center for Disease Control and Prevention, Shenzhen, Guangdong 518055, China; ^3^ Gannan Medical University, Ganzhou, Jiangxi 341000, China; ^4^ Zhongshan Center for Disease Control and Prevention, Zhongshan, Guangdong 528400, China; ^5^ Shenzhen Prevention and Treatment Center for Occupational Diseases, Shenzhen, Guangdong 518020, China

**Keywords:** arsemic exposure, CCM3, genetic polymorphism, blood presssure

## Abstract

Epidemiologic study has suggested that arsenic exposure is positively related to increased blood pressure. However, the underlying mechanism concerning interaction between genetic polymorphisms and arsenic exposure remains unclear. In present study, within 395 Chinese, the effects of interaction between arsenic exposure and CCM3 gene polymorphisms on elevation of blood pressure were probed by multiple Logistic regression models after adjusting for confounding factors. Firstly, we found that serum arsenic was positively associated with blood pressure, cholesterol, glucose and C-reactive protein. Then, adjusted for confounding factors of age, gender, smoking, alcohol consumption, BMI and degree of education, arsenic exposure incurred the hazard of increased systolic pressure and diastolic pressure, with odds ratios (*ORs*) being 1.725 and 1.425, respectively. Distinctly, we found that interactions between rs3804610^*^ rs9818496, rs6784267^*^rs9818496, and rs3804610^*^ rs6784267 variant genotype can increase significantly risks of SBP. Additionally, interactions between rs9818496, rs3804610 and rs6784267 genotypic variantions and arsenic exposure boosted the hazard of increased systolic pressure, with *ORs* being 1.496, 1.496 and 1.312. In conclusion, our fingdings suggest that As exposure of population can assist CCM3 polymorphism in elevating SBP.

## INTRODUCTION

Cardiovascular disease (CVD) is a major cause of death worldwide and a lot of evidence indicates that environmental toxicant exposures can deeply influence on CVD risk. Arsenic (As) is widespread in the environment, studies showed that As exposure was associated with elevated risks of CVD, even at lower levels of exposure (<100 μg/L) [1–3]. The legal limits in China on arsenic threshold was 10 μg/L, and it is recognized that exposure to arsenic levels greater than 50 μg/L in drinking water is considered high, whereas exposure to arsenic level less than 50 μg/L in drinking water is considered low to moderate. Recent study has linked arsenic exposure to increased preclinical indicators of CVD risk, including carotid intima media thickness, hypertension, and plasma markers of oxidative stress and endothelial dysfunction [4–7]. Hypertension can cause a series of health problems including heart failure, peripheral vascular disease, renal impairment and visual impairment through damage to endothelial cells. Recently, prehypertension was considered to be a risk factor for hypertension and CVD development [8]. High blood pressure (BP) is a primary risk factor for cerebrovascular disease and coronary heart disease. High BP contributes to about 7.5 million deaths every year worldwide. Several epidemiologic studies have found increased prevalence of hypertension or increased systolic and diastolic blood pressure levels among residents whose water supply has high level of arsenic (>100 μg/L) [1, 4, 6, 9, 10].

However, whether genetic polymorphism has influence over the relationship between As exposure and BP remains to be investigated. Exposure to arsenic increases BP by influencing cellular pathways and mechanisms [1, 6], and it can also induce oxidative stress, inflammatory responses, endothelial NO production, and alter gene expression [11–13]. As exposure has also been related to increases endothelial dysfunction [5, 14] which may increase the risk of hypertension, atherosclerosis and other CVDs. Recent study supported that the interaction between As exposure and *ICAM1*(intercellular adhesion molecule-1)*, VCAM1*(vascular cell adhesion molecule-1), and *CYBA* can influence the risk of CVD [7, 15, 16]. Cerebral cavernous malformations (CCMs) are vascular lesions characterized by abnormally enlarged capillary cavities, affecting the central nervous system. CCMs can be attributed to mutations in three different genes: CCM1, CCM2, and CCM3. CCM3 is also referred to as programmed cell death 10 (PDCD10) owing to the proapoptotic role *in vitro*. CCM3 is broadly expressed, including in neuronal and endothelial cells [17, 18]. CCM3 is an adaptor protein that can bind to a variety of proteins and protein complexes [19], which involved in cell to cell adhesion such as paxillin [20]. CCM3 can also bind to the germinal center kinase III (GCKIII) family of protein kinases, which is important to vascular development and for cell survival after oxidative stress [21]. CCM3 also plays a fundamental role in vascular development and angiogenesis. Endothelia-specific deletion of CCM3 in mice led to embryo death caused by angiogenesis defects and disruption of vascular integrity [22]. Cardiac and cranial vascular defects were also found in CCM3-deficient zebrafish [23]. These data indicated that CCM3-deficiency impairs vascular development/maturation. Genetic polymorphisms of CCM3 could influence cardiovascular-related physiological responses. However, the roles of CCM3 genetic polymorphisms on the relationship between As-exposure and increased BP are still poorly understood.

To test the hypothesis, we examined first the effects of As exposure on the indicators of CVD in two cohorts with different exposure level. Then, to investigate underlying effects of interaction between arsenic exposure and CCM3 polymorphisms, we conducted analyses of multiple Logistic regression to select and adjust the risk factors of high BP. Our analyses revealed interactions between CCM3 polymorphisms and arsenic exposure boosted the hazards of increased systolic pressure in south chinese, and our results demonstrate that arsenic exposure assist CCM3 polymorphisms in elevating blood pressure.

## RESULTS

### Arsenic exposure increased early risk markers of cardiovascular disease in south chinese population

As shown in Table 1, the > 4 μg/L blood As group has much higher Urinary As, but there was no significant difference between them (*p*> 0.05). 0-4 μg/L blood As group was matched with > 4 μg/L blood As group in terms of age, gender, body mass index (BMI), smoking status, drinking status, and education (all *p*> 0.05). The > 4 μg/L blood As group has much higher Systolic blood-pressure, Diastolic blood-pressure, Blood sugar, Cholesterol, C-reactive protein than 0-4 μg/L As blood group (all *p*< 0.05).

**Table 1:** General characteristics and early risk markers of cardiovascular disease of the subject

Characteristic	0-4 μg/L(n=280)	> 4 μg/L (n=114)	*p*-value
General characteristics			
Urinary arsenic (μg/L)	14.87	16.47	0.291
Age	25.76±6.57	26.43±5.76	0.413
Gender, Male/Female(% male)	79.60%	82.60%	0.499
Smoking status, Yes/No(%Yes)	32.00%	29.70%	0.273
Drinking status, Yes/No(%Yes)	17.70%	19.80%	0.623
Education level			
Low	2.10%	1.80%	0.406
Medium	72.90%	66.70%	
High	25.00%	31.60%	
BMI (kg/m^2^, mean± SD)	20.78±3.5	20.77±3.52	0.977
Family history, Yes/No(%Yes)	20.12%	25.17%	0.223
early risk markers of CVD			
Blood sugar	4.18±1.17	3.74±1.09	**0.001**
Cholesterol	4.58±1.10	4.99±1.03	**0.001**
Low density lipoprotein	1.99±0.87	1.98±0.95	0.9
High density lipoprotein	1.70±0.51	1.62±0.34	0.762
Triglyceride	1.07±0.79	0.97±0.37	0.224
C-reactive protein	5.82±5.39	7.02±4.72	**0.04**
Heart rate	76.84±12.20	74.93±10.80	0.148
Systolic blood-pressure(mmHg)	120.20±12.20	123.38±12.03	**0.019**
Diastolic blood-pressure(mmHg)	76.63±9.36	79.02±9.14	**0.022**
Electrocardiogram, Normal/Abnormal(%Normal)	78.03%	75.68%	0.62

### Predictors of increased SBP or DBP

Multivariate Logistic regression analysis was conducted by setting blood pressure as the dependent variable, and setting blood arsenic and urinary arsenic exposure, CCM3 gene polymorphism rs9818496, rs3804610, and rs6784267 as independent variables. After adjusting for age, gender, smoking, alcohol consumption, BMI and degree of education, the results showed that >4 μg/L of blood arsenic group had 1.725 (95%CI: 1.112-2.667) and 1.425 (95%CI: 1.012-2.161)-fold higher risk of SBP and DBP than 0-4 μg/L of blood arsenic group, respectively, but the urinary arsenic was not the risk factor. BMI (18.5-25) and higher education level were the protective factors respectively for SBP and DBP, the *OR* were 0.601(95% CI: 0.358∼0.983) and 0.592 (95% CI: 0.387∼0.906) respectively,as shown in Table 2.

**Table 2:** Multivariable Logistic regression analysis the risk factors of the blood

Variable		Systolic blood-pressure				Diastolic blood-pressure			
		*β*	*S.E*	*P*	*OR(95%CI)*	*β*	*S.E*	*P*	*OR(95%CI)*
Blood As	0-4								
	>4	0.57	0.263	**0.027**	**1.725(1.112∼2.667)**	0.429	0.288	**0.045**	**1.425(1.012∼2.161)**
Urine As	0-12.8								
	>12.8	0.317	0.289	0.396	0.719(0.346∼1.361)	0.203	0.318	0.465	0.802(0.397∼1.417)
rs9818496	CC								
	CT/TT	0.423	0.204	0.081	1.524(0.876∼2.362)	0.154	0.195	0.621	1.148(0.517∼1.878)
rs6784267	CC								
	CT/TT	0.043	0.256	0.793	1.040(0.646∼1.747)	0.266	0.301	0.272	1.335(0.812∼2.207)
BMI	<18.5								
	18.5-25	-0.472	0.232	**0.047**	**0.601(0.358∼0.983)**	-0.542	1.172	0.361	0.801(0.467∼1.341)
	25-30	-0.074	0.423	0.861	0.935(0.432∼2.138)	-1.136	1.164	0.835	0.923(0.430∼1.981)
	>30	0.478	1.978	0.685	1.618(0.151∼15.768)	0.135	1.243	0.401	2.758(0.265∼28.733)
Education	Low								
	Medium	-0.572	0.746	0.442	0.564(0.131∼2.435)	0.788	0.822	0.338	2.199(0.439∼11.001)
	High	-0.323	0.761	0.671	0.724(0.163∼3.216)	-0.532	0.208	**0.019**	**0.592(0.387∼0.906)**
Drinking status	No								
	Yes	0.001	0.252	0.992	1.001(0.612∼1.635)	0.361	0.249	0.146	1.432(0.882∼2.329)
Smoking status	No								
	Yes	0.032	0.225	0.899	1.029(0.670∼1.589)	0.054	0.224	0.805	1.058(0.681∼1.633)
Blood sugar		0.083	0.561	0.136	2.307(0.768∼6.928)	0.735	0.545	0.177	2.086(0.717∼6.073)
Cholesterol		-0.103	0.343	0.764	0.902(0.461∼1.767)	0.462	0.311	0.137	1.587(0.846∼2.917)
LDL		-0.552	0.475	0.246	0.576(0.227∼1.462)	-0.713	0.45	0.113	0.490(0.203∼1.184)
HDL		-0.766	0.712	0.282	0.465(0.115∼1.875)	-0.895	0.738	0.225	0.408(0.096∼1.736)
Triglyceride		1.05	0.565	0.063	0.857(0.944∼8.640)	1.013	0.532	0.057	2.573(0.970∼7.812)
CRP		0.017	0.039	0.669	1.017(0.942∼1.098)	0.076	0.04	0.057	1.079(0.998∼1.167)
Heart rate		0.004	0.02	0.82	0.996(0.965∼1.029)	-0.009	0.017	0.582	0.991(0.959∼1.024)

### CCM3 polymorphisms evelate BP in interactive manner

Firstly, to identify whether CCM3 polymorphism has roles in increased BP, we conducted a multivariate Logistic regression analysis. Here, blood pressure was setted as the dependent variable, with CCM3 polymorphism rs9818496, rs3804610, and rs6784267 being setted as independent variables. After adjusting for the confunding factors of blood arsenic and urinary arsenic, age, gender, smoking, alcohol consumption, BMI and degree of education, with *P*<0.05 as significant level, CCM3 polymorphism were not found to be associated to **SBP or DBP.** The detailed results were shown in Table 2.

Further, to investigate effects of interactions of CCM3 polymorphisms on blood pressure, we conducted multivariate Logistic regression analyses. Similarly, blood pressure was setted as the dependent variable, with CCM3 rs3804610^*^rs9818496, rs6784267^*^rs9818496, and rs3804610^*^ rs6784267 variant genotype being setted as independent variables. After adjusting for the confunding factors, we found that interactions between rs3804610^*^ rs9818496, rs6784267^*^rs9818496, and rs3804610^*^ rs6784267 variant genotype can increase significantly risks of SBP. The *ORs* values were detailed in Table 3.

**Table 3:** The interaction between gene and gene in the change of blood pressure

Gene-gene	Genotype		Systolic blood-pressure		Diastolic blood-pressure	
			*OR(95%CI)*	*OR^a^(95%CI)*	*OR(95%CI)*	*OR^a^(95%CI)*
rs3804610^*^ rs9818496	TT	CC	1	1	1	1
	TT	CT/TT	0.931(0.425∼2.038)	0.902(0.461∼1.767)	0.802(0.528∼1.391)	0.782(0.498∼1.291)
	TC/CC	CC	1.037(0.638∼1.687)	1.005(0.603∼1.623)	1.332(0.806∼2.200)	1.257(0.697∼1.919)
	TC/CC	CT/TT	1.558(1.078∼2.289)	1.498(1.016∼2.260)	1.465(1.006∼2.133)	1.339(0.891∼2.112)
			***OR_int_*=1.272(1.043∼1.551), *P*=0.017**		***OR_int_*=1.152(0.085∼1.951), *P*=0.087**	
rs6784267^*^ rs9818496	CC	CC	1	1	1	1
	CC	CT/TT	1.157(0.717∼1.869)	1.148(0.703∼1.824)	0.856(0.618∼1.491)	0.793(0.513∼1.301)
	CT/TT	CC	1.518(0.941∼2.450)	1.432(0.881∼2.327)	1.057(0.683∼1.636)	0.923(0.431∼1.977)
	CT/TT	CT/TT	1.609(1.108∼2.345)	1.552(1.041∼2.324)	1.490(1.024∼2.168)	1.366(0.914∼2.041)
			***OR_int_*=1.461(1.172∼1.822), *P*=0.001**		***OR_int_*=1.172(0.083∼1.976), *P*=0.093**	
rs6784267^*^ rs3804610	TT	CC	1	1	1	1
	TT	CT/TT	1.157(0.717∼1.869)	1.148(0.703∼1.824)	0.856(0.618∼1.491)	0.793(0.513∼1.301)
	TC/CC	CC	1.518(0.941∼2.450)	1.432(0.881∼2.327)	1.057(0.683∼1.636)	0.923(0.431∼1.977)
	TC/CC	CT/TT	1.609(1.108∼2.345)	1.552(1.041∼2.324)	1.490(1.024∼2.168)	1.366(0.914∼2.041)
			***OR_int_*=1.461(1.172∼1.822), *P*=0.001**		***OR_int_*=1.172(0.083∼1.976), *P*=0.093**	

*OR^a^* were adjusted for age, gender, BMI, smoking status, alcohol use status, and education level, BG, TG, TC, HDL, LDL, CRP.

Thus, resultant findings showed that CCM3 did not indenpendtly influence the blood pressures, but elevate the blood pressures in interactive manner.

### CCM3 polymorphism assists as exposure in elevating blood pressure

Eventually, we analyzed the effects of interactions between CCM3 polymorphisms and As exposure on the increased blood pressures in analogous way. Notely, rs9818496, rs3804610, and rs6784267 variant genotype with > 4 μg/L blood As significantly increased risk of SBP, with *ORs* being 1.496 (95% CI: 1.149∼1.947), 1.496 (95% CI: 1.149∼1.947) and 1.312 (95% CI: 1.081∼1.593). The detailed results were shown in Table 4. These results suggested that As exposureof population can assist CCM3 polymorphism in elevating blood pressures.

**Table 4:** The interaction between gene and As exposure in the change of blood pressure

Gene site	Genotype	As	Systolic blood-pressure		Diastolic blood-pressure	
			*OR(95%CI)*	*OR^a^(95%CI)*	*OR(95%CI)*	*OR^a^(95%CI)*
rs9818496	CC	0-4	1	1	1	1
	CC	>4	1.567(1.312∼2.762)	1.618(0.526∼2.817)	1.301(0.572∼2.731)	1.324(0.717∼2.946)
	CT/TT	0-4	1.312(0.518∼4.723)	1.354(0.753∼2.574)	1.245(0.372∼3.381)	1.271(0.417∼3.842)
	CT/TT	>4	2.437(1.318∼4.483)	2.682(1.348∼5.547)	1.547(0.922∼2.581)	1.565(0.927∼2.876)
			***OR_int_*=1.496(1.149∼1.947), *P*=0.003**		***OR_int_*=1.308(0.874∼1.902), *P*=0.067**	
rs3804610	TT	0-4	1	1	1	1
	TT	>4	1.567(1.312∼2.762)	1.618(0.526∼2.817)	1.301(0.572∼2.731)	1.324(0.717∼2.946)
	CT/CC	0-4	1.312(0.518∼4.723)	1.354(0.753∼2.574)	1.245(0.372∼3.381)	1.271(0.417∼3.842)
	CT/CC	>4	2.437(1.318∼4.483)	2.682(1.348∼5.547)	1.547(0.922∼2.581)	1.565(0.927∼2.876)
			***OR_int_*=1.496(1.149∼1.947), *P*=0.003**		***OR_int_*=1.308(0.874∼1.902), *P*=0.067**	
rs6784267	CC	0-4	1	1	1	1
	CC	>4	1.453(1.002∼3.244)	1.461(0.872∼2.414)	1.352(0.836∼2.463)	1.399(0.856∼2.589)
	CT/TT	0-4	1.262(1.116∼2.784)	1.286(0.714∼2.234)	1.286(0.736∼2.363)	1.327(0.906∼2.398)
	CT/TT	>4	1.789(1.132∼2.976)	1.824(1.094∼2.435)	1.493(0.950∼2.463)	1.539(0.956∼2.979)
			***OR_int_*=1.312(1.081∼1.593), *P*=0.006**		***OR_int_*=1.277(1.055∼1.546), *P*=0.052**	

*OR^a^* were adjusted for age, gender, BMI, smoking status, alcohol use status, and education level, BG, TG, TC, HDL, LDL, CRP.

## DISCUSSION

Many prospective studies have confirmed a strong association between BP levels and risk of CVD [24, 25]. A number of study reported that the prevalence of hypertension was positively related to As exposure [26–31]. In a prospective study, maternal urinary arsenic during pregnancy was correlated with BP in children at 4.5 years of age [32]. In this work, we investigated the influences of interactions of CCM3 polymorphisms and with As exposures on the blood pressures in south chinese. We found that, besides interactions of genetic polymorphisms implicating in the processes of increased blood pressures, CCM3 contributed to increased blood pressures through assisting the effects of As exposure. In our study, participants with >4μg/L blood As level had 1.733 (95%CI: 1.119∼2.684) and 1.476 (95%CI: 1.104∼2.153) fold higher risk of SBP and DBP, respectively. Prehypertension is associated with an about 3-fold risk of developing hypertension and roughly 2 times of cardiovascular events compared with normal BP [33]. Li, et al. found that the highest tertile had 1.69 (95% CI: 1.03∼2.78) fold higher risk of hypertension when compared with the lowest tertile of urinary arsenic concentration amongst Chinese population, where the lowest tertile had exposure to 0-0.65 mg/L of As through drinking water [34]. However, our results showed that the urinary arsenic is not the more sensitive marker in our study, but the blood arsenic is can increase the risk of blood pressure. This may be due to the arsenic in blood is recent arsenic exposure markers and the urinary arsenic is long-term arsenic level, but the change of blood pressure is very sensitive in influence factor. Arsenic exposure has been correlated to increased inflammation and endothelial damage [5, 14, 35], suggesting that arsenic may play partly by inducing endothelial dysfunction, atherosclerosis, and pathologic vascular remodeling. In our study, we found that arsenic was positively association with cholesterol, glucose and C-reactive protein (CRP), consistently with previous study.

CCM3 plays a fundamental role in vascular development, and CCM3 is an adaptor protein that can bind to a variety of proteins and protein complexes to regulate many important cell processes such as cell survival and cell death after oxidative stress [19, 20, 36]. In our previous work, we found that arsenic alters the expression of miR-425-5p and its target, CCM3 *in vitro* and *in vivo*, which leads to arsenic-induced anti-angiogenesis in HUVECs. These findings suggested that CCM3 participate in arsenic induced vascular cell development and endothelial dysfunction [37]. In this work, we investigated the association between CCM3 3 SNPs and BP change, and found that alone SNP did not increased the risk of SBP and DBP, but interactions between rs3804610^*^rs9818496, rs6784267^*^rs9818496, and rs3804610^*^ rs6784267 variant genotype increased the risk of SBP, and the interactions between rs9818496 and rs3804610 variant genotype with >4 μg/L As level significantly increased risk of SBP. Therefore, CCM3 may represent a novel susceptibility gene for hypertension in population with arsenic exposure. The variant genotype of CCM3 may impair vascular development and CCM3 is an adaptor protein which is regulated by stimulation, such as arsenic, so the mutation of CCM3 can influence vessel function and increase the risk of blood pressure, but the detailed molecular mechanism is not well understood. We need demonstrate the mechanism and find more related SNPs of CCM3 in arsenic exposure population in the further.

The strength of this study includes the low prevalence of hypertension medication in the population, so we were able to estimate BP changes in the relative absence of intervention. Furthermore, we assessed the interaction between As exposure and CCM3 genetic polymorphisms on blood pressure. It is an advantage in the ethnic homogeneity of the population, as it reduces bias from population stratification. On the other hand, our study also has limitations. First, our study is a cross-sectional study in which we measured blood arsenic, blood pressure, and preclinical indicators of CVD at the same time and it is not possible to determine whether differences in blood pressure and preclinical indicators of CVD followed arsenic exposures. Our subject is primarily middle-age males, which may limit generalizability of study findings. Second, likely other genes or SNPs play a role in increasing BP and that other genetic polymorphisms may interact with As exposure. Third, other risk factors, such as physical exercise and nutrition et al, could have potential impacts on blood pressure across life. Considerration of these factors, we included smoking status in our model as a possible confound factor. We left aside dietary factors such as high-fat diet or sodium intake in this study, because other studies have indicated that although these factors are associated with increases in BP, arsenic cannot interplay with dietary factors to raise longitudinal blood pressure in this population [38]. Fourth, our subject is different from elsewhere Chinese population and may have varied underlying risk factors that could influence blood pressure changes, such as diverse lifestyle, dietary styles and other surrounding factors. Despite these differences, serious of studies have argued that arsenic exposure play an important role in incidence and mortality of CVD, as well as blood pressure changes in populations.

In Conclusion, our fingdings suggest that As exposure of population can assist CCM3 polymorphism in elevating SBP. Our findings support the concept that genetic variants by themselves may not substantially influence disease risk, but in concert with environmental exposures, they may increase the risk of disease.

## MATERIALS AND METHODS

### Study design and participants

The present study was approved by Medical Ethics Committees of Sun Yat-sen University and this study was conducted in accordance with the guidelines of Medical Ethics Committees. Between April 2013 and October 2014, we selected 395 participants from electronic product workers in Zhongshan (Guangdong, China). These individuals met the following inclusion criteria [39]: 1) without any self-reported diseases, such as cardiopulmonary diseases, chronic inflammation, kidney diseases, and cancers; 2) without any self-reported medication use in the preceding three months; 3) without any significant changes in their occupational experiences, living environment, and lifestyle (such as smoking and drinking) in the past one year; 4) can provide enough biological materials for measurement of SNPs and blood arsenic. After written informed consent was obtained from each participant, trained interviewers performed a questionnaire to collect personal information such as general characteristics, lifestyle, medical history, medication usage, occupational, and environmental exposure experiences. After physical examination, each participant donated and about 2 mL EDTA anti-coagulated overnight fasting venous blood.

### Measurement of blood and urine arsenic and CCM3 single nucleotide polymorphisms (SNPs)

Venous whole blood samples collected at baseline were analyzed for arsenic concentration by inductively coupled plasma mass spectrometry (ICP-MS). The urinary samples were analyzed for arsenic concentration by hydride generation atomic flurescence spectrometry. Any DNA samples with a concentration < 40 ng/μL, and a 260 nm/280 nm ratio outside of the range of < 1.6 to ≥ 2.1 (measured by eppendorf) were excluded. Genotyping of rs9818496, rs3804610, and rs6784267 was performed using imLDR method by Shanghai Tian Hao Biological Science and Technology Co., Ltd (Shanghai, China).

### Statistical analysis

We conducted data analysis using SPSS, version 13.0 (SPSS Inc., Chicago, IL, USA). We first performed descriptive analysis to compare the distribution of demographic between low As level and high As level, using Chi-square tests for categorical and student's t-test for continuous variables, respectively. Statistical significance refers to *p*<0.05. In logistic regression analysis, participants were categorized according to baseline SBP (i.e. <120 mm Hg, normal or ≥120 mm Hg, pre-hypertensive to hypertensive) and by baseline DBP (i.e. <80 mm Hg, normal or ≥80 mm Hg, pre-hypertensive to hypertensive). In dominant genetic models, mutation genotypes CT/TT (CT/CC) were intergrited and compared to wild genotype CC (TT).

We performed multiple Logistic regression models to evaluate the odds ratio (OR) and the 95% confidence interval (95% CI) of the risk of BP change of blood As levels and 3 SNPs after adjustment for the confounders including age, gender, body mass index, smoking status, drinking status, education level, blood sugar, cholesterol, low density lipoprotein, high density lipoprotein, triglyceride, C-reactive protein.

For the interaction effect of two genetic variant, we used Logistic regression multiplicative model to estimate the odds ratio (OR) and the 95% confidence interval (95%CI) of the risk of BP change by differ genotypes with adjustment for potential confounders.

For the interaction effect of blood As levels and a genetic variant, we used Logistic regression multiplicative model to estimate the odds ratio (OR) and the 95% confidence interval (95% CI) of the risk of BP change by two of blood As levels and differ genotypes with adjustment for potential confounders.

## Abbreviations

As: arsenic
BP: blood pressure
CRP: C-reactive protein
SBP: systolic pressure
DBP: diastolic pressure
*OR*: odds ratio
CVD: Cardiovascular disease
BMI: body mass index.
